# Practical Chemistry for Students and Physicians—Chapter Second*Copyright secured.

**Published:** 1883-08

**Authors:** 


### PRACTICAL CHEMISTRY FOR STUDENTS AND PHYSICIANS—CHAPTER SECOND*

(Continued from July Register, Page 525.)

*Notation of Chemical Elements.*—Chemistry owes to Berzelius the use of abbreviations—symbols and numerals—

*Copyright secured.

by which its language is so much simplified. He gave to each atom the first letter of its Latin name, to be its symbol or representative; as O, for oxygen, and H for hydrogen.

Where several names begin with the same letter, a second letter is added to the first to distinguish the element, the single letter being always reserved for the most important of the group; as, C for carbon, Ca, calsium, and Cl, chlorine. Carbon, being the leading element, takes the initial letter.

*Notation of Quantivalence.*—The degree of quantivalence is indicated by the Roman numerals, except the first three, it being most convenient to distinguish them by simple scores or marks; as, in hydrogen H', designates both the atom and quantivalence of hydrogen; O'' the atom and quantivalence of oxygen; N''' the atom and quantivalence of nitrogen. The quantivalence of all the other elements being expressed in Roman numerals, that of Fe, or iron, is both Fe'' or $Fe_2$ vi, according to the compound presented of that varying element. Carbon is Civ; sulphur is either S'', Siv or Svi, like iron; chlorine is either Cl', Cl''', Clv or Cl,vii, as already explained, and the same may be written of bromine and iodine.

*Latin names of the elements with their symbols and quantivalences and atomic weights.* Of the sixty-seven elements now known, thirty-nine or forty comprehend all that is important or practical to the medical student or pharmacist. The larger number of the remainder are mere scientific curiosities.

| *Monads:* | *Sym and Quantiv.* | *Atom Wgt.* |
|---|---|---|
| Hydrogen | H' | 1 |
| Fluorine | F' | 19 |
| Chlorine | Cl',''', v vii | 35.5 |
| Bromine | Br.',''' v vii | 80 |
| Iodine | I' ''' v vii | 127 |
| Litheum | L' | 7 |
| Potassium | K' '''v | 39 |
| Silver | Ag'''' | 108 |
| Sodium | Na'''' | 23 |

*Dyads:*

| | | |
|---|---|---|
| Oxygen | O" | 16 |
| Sulphur | S" iv,vi | 32 |
| Selenium | Se",iv,vi | 79 |
| Calsium | Ca",iv | 40 |
| Strontium | Sr",iv | 87.5 |
| Barium | Ba",iv | 137 |
| Magnesium | Mg" | 24 |
| Zinc | Zn" | 65 |
| Cadmium | Cd" | 112 |
| Cerium | Ce" | 92 |
| Mercury | Hg",($Hg_2$)" | 200 |
| Copper | Cu",($Cu_2$)" | 635 |

*Triads:*

| | | |
|---|---|---|
| Nitrogen | N',''',v | 14 |
| Phosphorus | P',''',v | 31 |
| Arsenic | As',''',v | 75 |
| Antimony | Sb''',v | 122 |
| Bismuth | Bi''',v | 210 |
| Boron | Bo''' | 11 |
| Gold | Au',''' | 196 6 |

*Tetrads:*

| | | |
|---|---|---|
| Carbon | C",iv | 12 |
| Silicon | Si.iv | 28 |
| Tin | Sn",iv | 118 |
| Aluminium | ($Al_2$)vi | 27.5 |
| Platinum | Pt",iv | 197 |
| Lead | Pb",iv | 207 |

*Pentads.*—No primary pentads used in medicine.

*Hexads:*

| | | |
|---|---|---|
| Iron | Fe",iv,vi | 52 5 |
| Magnesia | Mg",iv,vi | 55 |
| Chromium | Cr",iv,vi | 55 |
| Cobalt | Co",iv | 59 |
| Nickel | Ni",iv | 59 |

*Heptads:*—No primary heptads in use in medicine and pharmacy.

The student should here commit carefully to memory the names only of the above thirty-nine elements; afterwards the names of the elements, with their symbols; then the names with the symbols and quantivalence; and last, the names with the symbols, quantivalence and atomic weights. Having done this thoroughly at once, he will greatly facilitate his subsequent course.

*Functions of the Symbols.*—Each symbol has four functions. 1st. It indicates the name of the element, as H, hydrogen, and O, oxygen. 2d. It indicates one atom of the element; as, H stands for one atom of hydrogen, or O for one atom of oxygen. 3d. It represents one volume of the element; as Cl. represents not only the substance chlorine and one atom of that substance, but, also, one volume of chlorine. 4th. It indicates the atomic weight of the element; as N represents not only the substance nitrogen, one atom of nitrogen and one volume of nitrogen, but at the same time the atomic weight of nitrogen.

*Multiplication of the Atom.*—The atoms are multiplied by placing an Arabic numeral below and a little to the right of the symbol; as, $H_2$ indicates two atoms of hydrogen, and $Ba_4$, four atoms or two molecules of barium. The elemental molecules are all represented in this way: $K_2$, $N_2$, $Cl_2$, $O_3$, ozone, $O_2$ oxygen, $As_4$, $S_6$, a hexatonic molecule of sulphur.

*Multiplicaiion of Molecules.*—These are multiplied by enclosing their symbols in parentheses, and placing their numeral as before—below and a little to the right, as $(Bi_2)_2$' gives two molecules of bismuth; $(Hg_2)_3$' indicates three molecules of mercury.

*Notation of Compound Bodies or Molecules.*—This is quite as simple as that just explained of the elemtntal molocules. It has already been seen that a compound molecule is one that is made up of unlike atoms; as a molecule of calomel, HgCl. Such a molecule may contain only two atoms, or as some of the hydro-carbons, it may have forty or fifty; as $C_5$, $H_{24}$, one of the essential oils.

*Binary Molecules.*—A compound molecule having only two constituents, as NaCl, common salt, or $HgCl_2$, corrosive sublimate, is a binary for that reason. Many of the most important bodies in chemistry belong to this class; such as the chlorides of K, Na, Ag, Pt and Ca—KCl, NaCl, AgCl, $PtCl_4$ and $CaCl_2$; also the bromides and oxides of all the metals, as KBr, Fe O, NaBr., $Fe.I_2$ and KI.

Binaries are always recognized by the terminal *ide* of their names; as iodide, bromide, chloride and oxide, etc.

*The Formation of Binaries.*—It is plain that these are formed by the simple union of their constituent atoms, observing at the same time, the law of quantivalence, and a rule, agreed upon among chemists, before alluded to in connection with notation and nomenclature; viz: That the positive constituent or root shall be written before the negative. All this is illustrated in the following example: The formula or molecule of water is $H_2O$, constructed by writing the symbols of the two constituents, hydrogen and oxygen, in a single expression. But the quantivalence of hydrogen is univalent, while that of oxygen is bivalent; hence the hydrogen atom must be multiplied to fulfil the law of quantivalence, and the same constituent must precede the oxygen, because it is the more positive element of the two. $H_2O$ is, therefore, the formula for water, and not $OH_2$. Much less can it be HO or OH. The systematic reading of the formula is *hydrogen oxide*; the positive element still preceding the negative in the oral language of the science.

*Binary Nomenclature.*—It may be already seen that no other branch of human knowledge can boast a more admirable system of nomenclature than chemistry. It is only surpassed by that of pure mathematics.

We are indebted for its fundamental rule to Lavoisier, of France: *The name of every chemical compound is made up of the names of its constituents.*

*Rule First, for Binary Compounds.*—All binary molecules are formed by the union of two simple elements; one of which, by the law of electro-chemical relation before explained, must be positive and the other negative.

*Rule of Construction for Names of Binary Compounds.*—The name of every binary molecule is written or spoken by placing the name of the positive constituent first, and after it the name of the negative, having its last syllable changed to *ide.*

*Examples.*—The molecule of potassium iodide is, KI, a formula which conveys, by a glance, the information that that important substance is composed of but two constituents; that those constituents are the metal potassium and the non-metal iodine; and furthermore, that in writing or pronouncing the name, the positive metal comes first, and after it the negative iodine with its syllable ene changed to ide.

*Modification of the Rule for Writing the Names of Binaries.*—This follows from the mysterious variation already noticed in quantivalence, and can be easily applied. The positive constituent may, by the law of variation of quantivalence, combine in two or more proportions with the negative constituent. In that case the higher proportion is indicated by the terminal *ic*, and the lower by that of *ous*.

*Example.*—There are two proportions in which the metal mercury combines with the negative chlorine, HgCl and $HgCl_2$. The first, the lower, is named by the rule of variation mercur*ous* chloride, and the second, the higher proportion, mercur*ic* chloride. So that in calling for these drugs it will be correct to say of the first, "Give me calomel, the mercurous chloride," or in common chemical language, the chloride of mercury; and in better chemical language, mercury chloride. And of the second, corrosive sublimate, mercuric chloride, bichloride of mercury, mercury bichloride.

*The Use of the Prefixes Hypo and Per in Nomenclature.*—In some instances, after the discovery of the first and second proportions in the combination of a positive with a negative constituent, a lower one than the first, and a higher than the second one is brought to light.

In this case, the proportion lower than the *ous*, is designated by *hypo*—"under"—and the one higher than the *ic* by *per*—meaning "above."

*Examples.*—A lower proportion than the *ous* has been detected in the union of chlorine and oxygen; $Cl'_2O$. This, then, is hypochlorous oxide; $Cl_2'''O_3$, being chlorous oxide.

But a higher than the *ic* compound of the same elements has also been discovered, $Cl_2viiO_7$. Hence this is the perchloric oxide; $Cl_2vO_5$ being the chloric oxide. These changes are obviously due to the variable quantivalence of the positive chlorine; and its indices of quantivalence in the several formulas are given to show it.

There is, also, a hyposulphurous oxide, S''O, but no per oxide of that element; and a hyponitrous oxide, $N_2$'O, without a pernitric oxide of nitrogen.

If the student will keep these facts in view when the consideration of acids, bases and salts come up, their nomenclature will give him no trouble whatever.

*Multiplication of Molecules or Formu'as.*—Molecules as well as atoms are frequently multiplied. This is done by placing a larger numeral than that used in the multiplication of the latter before the formula, as $2H_2O$, $3SO_2$, or $4NaCl$—meaning two molecules of water, thrice the molecule of sulphurous oxide, and four times the quantity of sodium chloride.

The same operation is sometimes indicated by the parenthesis and the small numeral, as in the multiplication of the atoms; thus $(H_2O)_2$, $(SO_2)_3$ and $NaCl)_4$,

*Rules for the Construction of Binary Molecules or Formulas when the Quantivalence of the Atoms is the Same or Different.*—When the quantivalence is the same in both, the rule is simple enough. *They unite in the proportion of* 1 *to* 1.

*Example.*—Na and Cl have the same univalent quantivalence; they therefore combine, one atom of each, to form a molecule of common salt, NaCl.

When the quantivalences differ, the rule is not so simple as the first, but plain and easy of application. When the combining atoms have different quantivalences they unite in the proportion of numbers obtained by dividing the least common multiple of their quantivalences by the quantivalence of each.

*Examples.*— H is univalent; O is bivalent; in order, therefore, to their mutual saturation in a compound, H must supply double the number of atoms of the oxygen. This is sufficiently plain without any calculation in so simple a case; but when higher quantivalences are involved it is often convenient to make the calculation according to the rule.

The least common multiple of the quantivalences of H and O is $1x2=2$. Then $2\div1=2$, gives the number of atoms to be supplied by the hydrogen; and 2÷2=1 the number to be supplied by the oxygen. The formula of water must, therefore, be written $H_2$ O.

Again, Cl is sometimes a heptad, and when thus written with bivalent oxygen, in what proportions of each will they form a molecule of perchloric oxide? The least common multiple is 7×2=14. Now, 14÷7=2, gives the saturating chlorine atoms, and 14÷2=7 the corresponding atoms of oxygen. Hence the molecule is $Cl_2O_7$.

These same laws are used in formulating acids, bases and salts, and on the same ground of varying quantivalences.

The way is now open for the special consideration of such characteristics of the elements as are important, and of the compounds formed by their unions with each other.

